# Non-Verbal Communication in Nursing Home Settings

**DOI:** 10.3390/healthcare14050614

**Published:** 2026-02-28

**Authors:** Zunera Khan, Miguel Vasconcelos Da Silva, Daniel Kramarczyk, Lise Birgitte Holteng Austbø, Martha Therese Gjestsen, Ingelin Testad, Clive Ballard

**Affiliations:** 1 Institute of Psychiatry, Psychology and Neuroscience, King’s College London, London SE5 8AZ, UKdaniel.kramarczyk@kcl.ac.uk (D.K.); 2 SESAM—Centre for Age-Related Medicine, Stavanger University Hospital, 4068 Stavanger, Norway; 3 College of Medicine and Health, University of Exeter, Exeter EX1 2LU, UK

**Keywords:** dementia, non-verbal communication, nursing homes, nursing home residents, dementia care

## Abstract

**Background:** People living with dementia in nursing homes commonly experience progressive impairments in cognition, communication, and functional ability, contributing to neuropsychiatric symptoms and reduced quality of life. As verbal communication declines, non-verbal communication (NVC) including facial expressions, gestures, eye contact, posture, and touch becomes increasingly important for maintaining meaningful interactions. **Objectives:** This study aims to explore current NVC practices between nursing home staff and residents living with dementia. **Methods:** A mixed methods, cross-sectional design was employed. NH staff completed an anonymous online questionnaire consisting of 13 items assessing NVC use and demographic characteristics. Quantitative items were rated using Likert scales, and qualitative responses were analysed using Giorgi’s phenomenological approach. **Results:** Quantitative findings showed that residents most frequently relied on facial expressions, reported as used very often in 24 of 33 NHs, followed by eye contact in 17 NHs and touch in 16 NHs. NH staff also reported extensive use of NVC during care interactions, particularly facial expressions (very often in 79% of NHs), eye contact (82%), and hand gestures (76%). Qualitative findings underscored the central role of NVC in interpreting residents’ needs, fostering emotional connection, and managing behavioural and psychological symptoms of dementia through subtle cues, visual prompts, and individualised strategies. **Conclusions:** Overall, the findings demonstrate that NVC is a fundamental component of communication and care delivery in dementia settings and highlight the need for structured training interventions to support staff in recognising and responding effectively to non-verbal signals.

## 1. Introduction

Worldwide, over 45 million people live with dementia (PlwD), including more than 850,000 in the UK, and this number is projected to reach 75 million by 2030 [1,2,3]. Approximately one-third of people with dementia reside in NH, particularly in the UK, Western Europe, and the United States, and around 80% of nursing home (NH) residents have either dementia or severe memory problems [3].

People living with advanced dementia have complex care needs, including prominent neuropsychiatric symptoms (NPS), which often lead to a reduced quality of life (QoL), especially in NH settings [4,5]. Communication impairments further compound these challenges, making it difficult for residents to express needs and maintain meaningful interactions.

Person-centred care interventions have demonstrated benefits in managing NPS and improving QoL, but these effects appear largely limited to individuals with mild to moderately severe dementia who retain verbal communication skills [6]. Recent evidence indicates that impairments in non-verbal communication (NVC) are independently associated with the severity of NPS, proxy-reported QoL, and agitation levels [7]. Optimising both verbal and NVC is therefore critical for fostering better social interactions and supporting NH residents.

Despite its potential, NVC remains relatively under-researched in dementia care [8]. Theoretical models of person-centred care, such as Kitwood’s concept of personhood, highlight the central role of attuned relational communication for supporting well-being in dementia [9]. Communication theory similarly emphasises the importance of non-verbal behaviours such as gaze, gesture, posture, and facial expression as verbal skills decline [10].

This study addresses these gaps by providing empirical evidence on how NVC is used between residents with dementia and NH staff, an area that remains under-researched. Existing work is dominated by small, single-site qualitative studies, leaving little systematic data on everyday NVC practices across multiple homes [11,12]. By integrating a quantitative approach to NVC patterns with phenomenological analysis of staff experiences, this study offers mixed methods examinations of real-world NVC in dementia care. The study helps towards informing targeted communication training within dementia care for NH staff.

### Aims

The overall aim of this study is to examine the use of NVC between NH residents living with dementia and NH staff.

**Specific Objectives**

- Identify the most commonly used NVC cues displayed by NH residents during routine care and social interactions.
- Identify the NVC strategies most commonly used by NH staff to interact with residents.
- Explore how NH residents use NVC during episodes of behavioural symptoms.
- Examine variations in NVC practices across NH characteristics, including care facility, size, and type of care.

## 2. Materials and Methods

The current study uses a mixed methods, cross-sectional observational design to examine NVC cues used by NH residents and NH staff.

### 2.1. Nursing Home Recruitment

NHs from the Care Home Research Network (CHRN), King’s College London, were invited via email to participate in a brief, anonymous online questionnaire. The invitational email included study information and a link to the questionnaire, which NH staff accessed independently to complete the self-report survey. Reminder emails were sent weekly by the CHRN team. Researcher contact details were provided in the participant information sheet to enable participants to seek clarification or ask questions. Ethical clearance was obtained from King’s College London Research Ethics Office (Ethical Clearance Reference Number: LRS-20/21-21632). Participating NH staff provided consent online prior to completing the anonymous questionnaire. There are different types of nursing home care facilities in the UK. The types of nursing home facilities may include homes (without nursing staff), residential homes (without nursing staff), nursing homes, assisted living (independent living with no care staff on site).

### 2.2. Questionnaire

The questionnaire included 13 questions on NVC and NH demographics. NH staff provided information on communication cues used between residents and staff during instrumental care and social activities. Study data were collected though an online self-report questionnaire via the Microsoft Forms platform (Study of Non-Verbal Communication in Dementia in Care Home Settings, https://forms.office.com/Pages/ResponsePage.aspx?id=FM9wg_MWFky4PHJAcWVDVtxw4u2XLelDqtPE-th9zQdUOEdGTkJJMFEzWFBTUkRGUTVJN0pBQlYySi4u, accessed on 23 February 2026).

A mixed methods approach [13] was taken to collect both quantitative data and qualitative data. NH staff were requested to report on the most commonly used non-verbal cues by their residents and NH staff for interaction during the delivery of care and social activities. The NH staff were also required to report on commonly used non-verbal cues by NH residents during periods of increased behavioural symptoms. Other key information collected through the questionnaire included NH demographics (including type of care setting, size and care speciality).

- What are the most commonly used non-verbal signals observed in your residents living with dementia?
- What non-verbal signals do you use most commonly to interact with your residents with dementia?

In parallel, the qualitative data (with an inductive approach), was collected using open text responses by NH staff to describe the use of NVC by NH residents and staff. The NH staff reported the following qualitative questions through a self-reported survey in written form:

Can you describe how do you use non-verbal communication, when one of your residents may express behavioural symptoms and during delivery of care on day-to-day basis?

What are the most common ways of communicating by your residents living with dementia (e.g., using non-verbal communication signals and words)?

### 2.3. Data Analysis

#### 2.3.1. Quantitative Analysis

Study data were analysed using Stata 17 statistical software. Descriptive statistics were presented for sample characteristics (NH size, type of care, type of care setting) with percentages. Key trends were noted as percentages for the use of most commonly used non-verbal signals observed by NH residents and staff. Two questionnaire items were analysed to quantify NVC, (i) reporting the NVC cues most commonly used by residents, and (ii) reporting the cues most commonly used by staff. Each question contained eight Likert-type items (Never–Very often). For each respondent, two composite NVC scores were calculated:

(1) A mean frequency score (average Likert value across the eight items), reflecting overall intensity of NVC use;
(2) A diversity score, defined as the number of NVC modalities rated “Often” or “Very often”, reflecting the breadth of NVC behaviours used.

The two questions were checked for internal consistency using Cronbach’s alpha. Internal consistency of the NVC items was acceptable, with a Cronbach’s alpha of 0.82 for resident NVC (Q14) and 0.78 for staff NVC (Q17).

Statistical significance was set at *p* < 0.05 (two-tailed). A Spearman correlation analysis was conducted to determine whether the demographic data of the NH (number of beds, type of care and care setting type) was associated with differences in the frequency of NVC used by residents and staff. In addition, Kruskal–Wallis tests were used to assess whether NVC scores differed across care home setting types (care, nursing, residential). Post hoc comparisons were conducted using Mann–Whitney U tests for each care-type category (providing vs. not providing that care), and *p*-values were adjusted for multiple comparisons using the Benjamini–Hochberg false discovery rate (FDR). Statistical significance was set at *p* < 0.05 (two-tailed). For post hoc analyses, FDR-adjusted *p*-values (pFDR) were used to determine statistical significance.

#### 2.3.2. Qualitative Analysis

The qualitative data were analysed using systematic text condensation techniques, with Giorgi’s psychological phenomenological analysis [14], which has been reliably applied in research in recent years, utilising basic elements of Giorgi’s approach [15,16]. Open text responses, given by NH staff, were read by the research team (ZK, IT, MVS, LB, MTB), obtaining an overall impression from each given response. Once the responses were read several times, the research team came to an agreement on preliminary main themes for the responses, which led to the identification of meaning units and classification into final codes. The research team discussed the final codes of the meaning units with the decontextualised material. The codes and meaning units were then regrouped and shuffled continuously to create subgroups for each code group, with consideration of important key messages and meaningful information picked from responses. Condensation and abstraction of the meaning units within each code and artificial quotes were subsequently attained. Finally, data were re-contextualized into an analytical text with a category heading. Artificial quotes from the third step were revised into a longer description of each category and constructed with a quote which appropriately represented the category.

## 3. Results

### 3.1. Nursing Home Care Facilities Demographics

A total of 225 nursing homes were invited to participate, of which 33 consented and completed the survey. Most respondents held managerial positions. An equal number of nursing homes (*n* = 11) and residential and care homes (*n* = 11) participated, alongside 10 residential homes and one other, unspecified care facility. Among the participating facilities, 30 out of 33 provided dementia care, 16 out of 33 catered for both mental and physical health needs, and 24 out of 33 provided care for general older adult populations.

The majority of the NH facilities presented a CQC rating of good (66%, *n* = 22), see Table 1.

### 3.2. Quantitative Findings

#### 3.2.1. What Are the Most Commonly Used Non-Verbal Signals Observed in Your Residents Living with Dementia (*n* = 33)?

Across 52% of the NHs surveyed (*n* = 33), residents living with dementia were reported to rely heavily on non-verbal communication (NVC). Facial expressions were the most frequently observed non-verbal signal, reported as used very often in 73% of NHs (*n* = 24) and often in 21% (*n* = 7). Touch was also commonly observed, reported as used very often in 49% of NHs (*n* = 16) and often in 33% (*n* = 11).

Eye contact was reported as occurring very often in residents in 52% of NHs (*n* = 17), often in 21% (*n* = 7), and sometimes in 21% (*n* = 7). Hand gestures were observed very often in residents in 36% of NHs (*n* = 12) and often in 49% (*n* = 16), while 15% reported sometimes or rarely (*n* = 5). Head nods were commonly observed, reported as very often in 39% of NHs (*n* = 13) and often in 42% (*n* = 14). Changes in pitch were observed very often in residents in 36% of NHs (*n* = 12) and often in 33% (*n* = 11), with 27% reporting sometimes or rarely (*n* = 9). Posture was noted very often in 24% of NHs (*n* = 8), often in 46% (*n* = 15), and sometimes or rarely in 30% (*n* = 10).

#### 3.2.2. What Non-Verbal Signals Do You Use Most Commonly to Interact with Your Residents with Dementia *(n* = 33)?

Across the 33 NHs, staff were reported to rely extensively on non-verbal communication (NVC) when interacting with residents living with dementia. Facial expressions were the most frequently used cue, reported as used very often in 79% of NHs (*n* = 26) and often in 18% (*n* = 6). Touch was also commonly used, with 67% of NHs reporting very frequent use (*n* = 22) and 30% reporting frequent use (*n* = 10).

Eye contact was reported as used very often in 82% of NHs (*n* = 27), with an additional 15% reporting frequent use (*n* = 5). Hand gestures were widely employed, reported as very often in 76% of NHs (*n* = 25) and often in 15% (*n* = 5). Head nods were utilised, with 67% of NHs reporting very frequent use (*n* = 22) and 18% reporting frequent use (*n* = 6).

Vocal pitch variation was reported as used very often in 64% of NHs (*n* = 21), often in 9% (*n* = 3), and sometimes or rarely in 27% (*n* = 9). The posture non-verbal cue was reported as used very often in 55% of NHs (*n* = 18), often in 22% (*n* = 7), and sometimes or rarely in 24% (*n* = 8).

#### 3.2.3. Associations Between NVC and Care Home Characteristics

Spearman correlations showed no significant associations between the number of beds and either resident NVC or staff NVC for both mean frequency and diversity scores (*p*-values ranged from 0.71 to 0.97; Table 2).

- Mean NVC score—the average Likert rating (Never–Very often) across the eight NVC cues, reflecting overall frequency of NVC use.
- Diversity score—the number of NVC cues rated “Often” or “Very often”, reflecting the breadth of different NVC behaviours used.

Kruskal–Wallis tests compared NVC scores across care settings (care homes, NHs, residential homes). No significant group differences were observed for either residents or staff (Table 3).

Mann–Whitney U (residents) tests compared homes that provided each care type versus those that did not. False Discovery Rate (FDR) correction was applied to control for multiple comparisons (Table 4 and Table 5).

Across all analyses, NVC use did not vary by home size or setting. However, homes providing Mental Health Condition care showed higher resident NVC scores; only resident NVC scores differed significantly by care type after FDR correction.

The NVC scales demonstrated good internal reliability (Q14: α = 0.82; Q17: α = 0.78).

### 3.3. Qualitative Findings

The purpose of the qualitative analysis was to gain insights into how NVC is employed by NH residents with dementia and their NH staff. NH staff shared valuable information on two primary fronts. Firstly, they reported on the common modes of communication observed among NH residents. Additionally, NH staff shared insights into how they employ non-verbal cues when addressing behavioural symptoms in residents with dementia. They also highlighted the crucial role these cues could play in the routine delivery of care, underscoring the importance of employing diverse communication channels to enhance understanding and support in daily interactions (Table 6).

- What are the most common ways of communicating by your residents living with dementia (e.g., using non-verbal communication signals and words)?
- In the use of NVC by NH staff in response to behavioural symptoms expressed by residents with dementia and during the daily delivery of care three main themes were identified: (i) Subtle non-verbal cues, (ii) visual prompts, and (iii) care strategies.

#### 3.3.1. Subtle Non-Verbal Cues

One theme that emerged was the frequent use of subtle NVC by staff in response to residents’ behaviours and during care. Both NH staff and residents with dementia used NVC reciprocally. This included soft tones, gentle touches like hand-holding or squeezing, open posture, eye level contact, gestures, sign language, and respecting personal space. Staff used these NVC cues to communicate, engage, and show affection to residents. The quotes below illustrate how NVC fostered both engagement and affection.

‘‘Gentle approach and a calming voice. Sometimes holding a handout may allow the resident to feel understand and they will hold your hand as a sign of thanks.’’ ‘‘Gentle nodding, touch, singing nursery rhymes to divert attention, hands movement, get attention by facing them directly. gentle touch.’’

‘‘Body Language/Eye Contact Approach Individuals from the front Facial Expressions Physical contact will give the person a sense of care and affection.’’

#### 3.3.2. Visual Prompts

NH staff used visual prompts to enhance communication and understanding during care, especially when residents exhibited behavioural symptoms. These prompts included pictorial cards, flashcards, demonstrations, and visual choices, such as showing a spoon before feeding or presenting clothing options. These strategies helped clarify tasks, foster engagement, and empower residents by giving them choices. Visual prompts often worked alongside non-verbal cues like gestures, pointing, and nodding to strengthen bonds and facilitate care, as illustrated in the quotes below.

‘‘Can talk and point to several items to try and distract or find out trigger point to pictures, toilet, parts of the body’’ ‘‘By giving them visual prompts, explaining always what we are going to do next, also showing options for example take 2 sets of clothing out of the of the wardrobe and they can choose which one they would like to wear that day. They non-verbally communicate it with pointing, nodding head, blinking’’.

#### 3.3.3. Care Strategies

Care strategies emerged as a key theme highlighted by staff, involving staffing adjustments, distraction techniques, and providing reassurance in care settings. Staffing strategies included adjusting personnel, returning later, and gradual task execution. Activities involved knitting, music, dance, colour use, and ensuring residents ate and drank before personal care. Staff offered reassurance by actively listening, using positive gestures, and maintaining a calm, comforting presence, fostering a supportive environment to ease residents’ anxiety. Non-verbal cues were also used to establish meaningful connections with NH residents. Examples of these strategies are shown in the quotes.

‘‘A particular resident does not like personal care. Before we start, we ensure he has had a drink & something to eat. Slowly preparing items that we would use for washing so that he can see them. A gentle squeeze of the hand to re-assure helps. And putting on gentle classical music without saying anything helps to calm him.’’

‘‘remain calm, offer a drink, facial expression with re assurance and comfort, eye contact, distract to another subject from current situation.’’ ‘‘Calm and soft pitch, distancing and leaving them for 10 min, they soon forget, tender touch looking into their eyes and pleading with them, so they understand.’’

## 4. Discussion

The study findings indicate that staff from the 33 responding NHs reported that residents and staff utilise diverse NVC cues to express themselves and engage with one another. Across quantitative and qualitative analyses, the most common cues included facial expressions, touch, eye contact and hand gestures (e.g., pointing, raising a hand, nodding or shaking the head), as well as aspects of voice and posture. Residents used these behaviours to convey emotions, signal basic care needs (such as touching the belly), express preferences and communicate willingness or reluctance to participate in activities, particularly during daily care routines and mealtimes. Staff drew on similar cues to offer reassurance, convey warmth and establish connection, using touch, an open posture, eye level contact and a modulated tone of voice. These findings support previous research indicating that NH residents retain NVC abilities even in the later stages of dementia, including gestures, eye gaze and facial expressions [7,12], and underscore the central role of these cues in everyday interactions in NH settings.

The additional statistical analysis further indicated that although NVC use was consistent across most NH characteristics (NH size, type of setting, type of care provided), homes providing specialist Mental Health Condition care reported significantly richer resident NVC, suggesting that behavioural care environments may cultivate or require greater use of non-verbal strategies.

A diminished quality of communication between NH residents and their NH could lead to misperception of intentions, an increased likelihood of perceiving risk or threat, and possibly to an increase in agitation, aggression, and other neuropsychiatric behaviours in NH residents with dementia in response [5]. By acknowledging the distinct non-verbal signals that residents exhibit, staff can enhance care, offering person-centred care aligned with each individual’s communication preferences.

The study also supports previous literature, which pinpoints the need to develop interventions that could cater to the needs of people with dementia in their severe stages of dementia. NH residents displayed a wide range of emotions throughout their journey, expressing both positive and negative moods. Positive moments involved joy, singing and dancing, whereas negative expressions included anger, aggression, sadness, and reluctance to participate in activities, accompanied by changes in appetite and agitation. In addition to non-verbal cues, staff benefit from using visual prompts to elicit responses from their residents. NH residents use a range of verbal expressions, such as brief sentences, single words, one-word signs, and straightforward language.

Taken together, staff members’ use of non-verbal strategies—including a gentle tone, reassuring touch, open posture and eye level contact—appeared to create a more supportive, person-centred communicative environment, enabling individuals with dementia who may not communicate verbally to convey their needs through non-verbal interactions during daily care and social activities.

The NH staff not only focused on enhancing communication strategies but also adjusted their care approaches to establish stronger connections and interactions with residents throughout their daily care routines and social engagements. NH staff introduced various activities, such as altering staff rotations, returning at a more convenient time, practising active listening, and engaging residents in pursuits like knitting, music, dance, colouring, and shared meals. The observation of residents engaging in dancing was noted as a potential indicator of a positive mood. It is worth mentioning that these activities extend beyond mere enjoyment, as studies [17] suggest that such interventions may yield positive behavioural outcomes for individuals residing in institutionalised care settings.

Intervention utilising non-verbal cues such as joint attention techniques has been shown to improve engagement and interaction in other people with learning disabilities [15,18]. Sharing attention through gestures, pointing, showing, and coordinating gaze between targeted objects and individuals could help in facilitating joint engagement, and constitute a turn-taking channel for mutually sustained engagement between NH residents and their NH.

Comparable conclusions have been reported previously supporting the use of NVC strategies when interacting with PlwD [11]. However, there is limited agreement on which outcomes best define effective supportive NVC. Given the evidence for the benefits of person-centred care, NVC highlights the need for future research to clearly specify how to implement NVC within person-centred care. International research also highlights communication as a key therapeutic target in dementia care, emphasising the need for system-wide education and multidisciplinary training to help health professionals recognise and respond to retained communication strengths, including non-verbal cues [11,18,19]. These international findings showcase the importance of our results and indicate that the patterns observed in UK care settings may reflect broader global challenges in dementia communication practice.

### 4.1. Impact

The current study suggests that non-verbal cues could serve as a therapeutic avenue to enhance the well-being of NH residents who may struggle to express themselves verbally. Building on this, there is a clear need for interventions that strengthen NH staff’s skills in NVC, particularly for residents in more advanced stages of dementia [6]. Implementing a holistic model with a central focus on communication between NH staff and residents has the potential to improve residents’ well-being and engagement in NH settings. In this context, a holistic model refers to an approach that considers the resident’s overall needs, including their sensory, emotional, cognitive, cultural, and physical wellbeing, recognising that communication occurs not only through speech but also through body language, facial expression, touch, posture, proximity, and other behavioural cues.

In practice, this could be operationalised through interventional research and structured training programmes aimed at improving NH staff’s skills in NVC. Such interventions may include practical modules which highlight the importance of NVC among PlwD, alongside strategies for addressing broader care needs. For instance, physical factors such as hearing impairment, sensory changes, pain, and fatigue can influence how residents express themselves non-verbally, while cultural values shape how gestures, touch, and eye contact are used and interpreted. Enhancing staff awareness of these influences is essential for accurate interpretation and responsive care.

Training could incorporate brief, applied components such as recognising common NVC cues among residents with dementia, using visual prompts to anchor shared attention, adapting tone and body orientation to create a sense of safety, and employing calming non-verbal approaches during behavioural symptoms. Additional elements could include role-play, reflective practice, and observational learning to help staff internalise skills and apply them consistently in daily care routines.

Embedding NVC training within a broader person-centred framework would support NH staff in recognising subtle non-verbal signals and responding to individual needs more effectively [6,7]. This approach would not only strengthen relationships between residents and staff but also promote autonomy, enhance engagement, and contribute to a deeper understanding of residents’ needs, ultimately improving the overall quality of care.

### 4.2. Limitations and Strengths

The current findings were obtained through online, anonymous mixed methods surveys completed by NH staff. Such surveys provide an accessible and efficient method of data collection, offering flexibility, reducing staff burden, and enabling timely insight into communication practices in care settings. By integrating quantitative and qualitative components, the study captured both numerical patterns and contextual nuances, strengthening the interpretation of findings and their relevance to real-world practice. The study utilised an exploratory theory-informed questionnaire to assess the use of NVC in care settings. Further research can be conducted to validate the questionnaire using a larger dataset.

A limitation of the study is the modest number of participating care facilities, which may constrain the generalisability of results. Respondents were predominantly individuals in managerial roles, potentially skewing findings toward organisational perspectives rather than everyday clinical practice. Although online data collection is efficient, it may lessen insight into the subtle dynamics of natural interactions within care settings.

Further research would benefit from recruiting a broader range of participants, particularly frontline clinical staff, to ensure a more representative understanding of communication practices. Incorporating direct observations or recordings of real-life care situations, as well as introducing and evaluating specific communication training interventions, would further strengthen the evidence base.

Given the exploratory nature of the study, post hoc power considerations indicate that the sample was sufficient to detect moderate-to-large effects in non-parametric analyses (Spearman’s *r* ≈ 0.45–0.50) at an alpha level of 0.05. Smaller effects may not have been detectable, and therefore, non-significant findings should be interpreted cautiously. The results are primarily intended to describe patterns of non-verbal communication and to generate hypotheses for future studies, rather than to provide definitive prevalence estimates or to support strong acceptance of null hypotheses.

Overall, these findings highlight the potential to develop a targeted, person-centred communication programme tailored for both NH residents and NH staff. Training that supports staff in adapting and refining their communication cues may better accommodate the needs of residents living with dementia and enhance the quality of care. Despite its limitations, the current study highlights the importance of non-verbal communication for enhancing care quality, guiding future research and practice in care settings.

## 5. Conclusions

NH residents and NH staff use a diverse range of NVC channels to interact, with NVC being the most frequently utilised strategy. When creating interventions to improve communication between staff and PlwD, a focus on NVC should be prioritised due to its prevalent use. Enhancing NVC skills among NH staff can lead to better identification and response to residents’ needs, reduce behavioural symptoms, and ultimately improve the quality of life for those in care.

## Figures and Tables

**Table 1:** Sample population and NH demographics.

	Percentages (Number)
**Responding role**	
Manager	57% (19)
Deputy Manager	9% (3)
Health Care Assistant	15% (5)
Nurse	3% (1)
Other unspecified	15% (5)
**Age of Staff reporting**	
20–30	9% (3)
30–40	30% (10)
40–50	27% (9)
50–70	33% (11)
**Type of Care facility**	
Nursing Home	33% (11)
Residential	30% (10)
Care home	33% (11)
Other unspecified	3% (1)
**Type of Care**	
Dementia	90% (30)
Mental Health	48% (16)
Physical Health	42% (14)
Old Age	72% (24)
**Number of beds**	
<30	18% (6)
30–100	75% (25)
>100	6% (2)
**Number of Staff**	
<30	30% (10)
30–100	57% (19)

**Table 2:** Spearman correlations between care home size and NVC scores.

Analysis	*r*	*p*	*n*
Resident NVC–mean score	0.067	0.712	33
Resident NVC–diversity score	−0.006	0.974	33
Staff NVC–mean score	0.059	0.743	33
Staff NVC–diversity score	−0.013	0.941	33

Spearman correlations indicated no significant associations between care home size (number of beds) and resident or staff non-verbal communication (NVC) scores (*p*-values above 0.05).

**Table 3:** Kruskal–Wallis tests comparing NVC scores across care home settings.

Analysis	*p*	Interpretation
Resident NVC–mean score	0.905	No difference
Resident NVC–diversity score	0.937	No difference
Staff NVC–mean score	0.930	No difference
Staff NVC–diversity score	0.994	No difference

No significant differences in resident or staff NVC scores were found across care home settings (*p*-values above 0.05).

**Table 4:** Mann–Whitney U tests comparing resident NVC scores by type of care.

Type of Care	*p*	*p*_FDR	Interpretation
Dementia	0.211	0.421	Not significant
Old Age	0.556	0.741	Not significant
Mental Health Condition	0.002	0.008	Significant difference
Physical Disability	0.898	0.898	Not significant

Homes providing mental health care reported significantly higher resident NVC scores (*p* = 0.002; *p*_FDR = 0.008). No other care types were associated with differences in NVC.

**Table 5:** Mann–Whitney U tests comparing staff NVC scores by type of care.

Type of Care	*p*	*p*_FDR	Interpretation
Dementia	0.270	0.359	Not significant
Old Age	0.108	0.215	Not significant
Mental Health Condition	0.017	0.070	Not significant
Physical Disability	0.359	0.359	Not significant

Homes providing mental health care showed a difference in staff NVC scores (*p* = 0.017), but this did not remain significant after FDR correction (*p*_FDR = 0.070). No other care types were associated with differences in staff NVC.

**Table 6:** Preliminary and sub-themes of NVC by NH staff.

Preliminary Theme	Sub Themes	Key Features
Subtle NVC	Staff/Resident’s NVC	soft pitch, soft tone, gentle touch; touch their hand, a gentle squeeze of the hand to re-assure him helps. open posture, relaxed posture, coming down to their level for eye contact, pointing, coming closer, looking directly at the person, sign language, respecting personal space, moving away, gesture; hand gestures, gentle nodding
Care Strategies	Staffing	Changing staff, coming back later, actively listening, doing things and preparing slowly
	Distraction and other activities	knitting, music, dance, colour, eating drinking before PC
	Reassurance	company them to make them know I am with them, making residents feel at ease and calm, stay with them to make them feel important
	Other care strategies	pleading and gentle persuasion, kind persuasion, avoiding risk
Visual Prompt	Visual cues	visual cues, pictorial cards, showing them what we are doing before the task; showing a spoon of food before offering it, showing choices, demonstration, showing cup, flash cards, point to picture, picture book, picture card, cue cards

## Data Availability

The datasets presented in this article are not readily available due to technical limitations. Requests to access the datasets should be directed to zunera.2.khan@kcl.ac.uk.
